# Internet-Based Computer Tailored Feedback on Sunscreen Use

**DOI:** 10.2196/jmir.1902

**Published:** 2012-04-30

**Authors:** Hein de Vries, Matti Logister, Gertruud Krekels, Frits Klaasse, Verina Servranckx, Liesbeth van Osch

**Affiliations:** ^1^School for Public Health and Primary Care (CAPHRI)Department of Health PromotionMaastricht UniversityMaastrichtNetherlands; ^2^Maastricht UniversityDepartment of Health PromotionMaastricht UniversityMaastrichtNetherlands; ^3^Catharina HospitalDermatology departmentEindhovenNetherlands

**Keywords:** Health communication, Computer tailoring, Web-based interventions, Internet

## Abstract

**Background:**

Skin cancer incidence rates signify the need for effective programs for the prevention of skin cancer and for helping skin cancer patients. Internet and computer tailored (CT) technology fosters the development of highly individualized health communication messages. Yet, reactions to Internet CT programs may differ per level of involvement and education level and remain understudied.

**Objective:**

First, we identified perceptions concerning sunscreen use in Dutch adults and assessed differences in differences between the general public and skin cancer patients, and between low and high educated respondents. Second, we assessed program evaluations of these groups about a new Dutch CT Internet-based program promoting sunscreen use, and potential differences between groups

**Methods:**

A cross-sectional research design was used. In total, 387 respondents participated and filled out an online questionnaire based on the I-Change Model assessing socio-demographics, history of skin cancer, sunscreen use, and beliefs about sunscreen use. The responses were fed into a computer program that generated personal tailored feedback on screen; next we assessed their program evaluations

**Results:**

Of the 132 patients, 92 were female (69.7%) and 40 were male (30.3%). In the general population (N = 225), 139 (54.5%) respondents were female and 116 (45.5%) were male. Men (50.9 years) were 8 years older than women (43.1 years). Most patients were diagnosed with basal cell carcinoma (N = 65; 49.2%), followed by melanoma (N = 28; 21.2%) and squamous cell carcinoma (N = 10; 7.6%); 22% (N = 29) did not remember their skin cancer type. Patients had higher knowledge levels, felt significantly more at risk, were more convinced of the pros of sunscreen, experienced more social support to use sunscreen, had higher self-efficacy, and made more plans to use sunscreen than respondents without skin cancer (N=255; all *P*’s< .01). Low (N=196) educated respondents scored lower on knowledge (*P*<.003) but made more action plans (*P*<.03) than higher educated respondents (N=191). The CT feedback was evaluated positively by all respondents, and scored a 7.8 on a 10 point scale. Yet, patients evaluated the CT program slightly more (*P*<.05) positive (8.1) than non-patients. (7.6). Lower educated respondents were significantly (*P*<.05) more positive about the advantages of the program.

**Conclusions:**

First, involvement with skin cancer was reflected in more positive beliefs toward sunscreen use in patients in comparison with non-patients. Second, the CT Internet program was well accepted by both patients and non-patients, and low and high educated respondents, perhaps because higher educated respondents were more knowledgeable about sunscreen use and skin cancer. Third, a pro-active approach as conducted in our study is very well suited to reach various groups of people and is more likely to be successful than a reactive approach

## Introduction

### Background

The Internet offers vast possibilities for health-communication efforts. Online health communication has the potential to reach large audiences, it can be operational at all times, and costs per visitor can remain low [1]. Internet access in the Netherlands has grown substantially from 16% in 1998 to 93% in 2009 [2]. Internet provides the possibility for reaching many people in the Netherlands [3]. Modern interactive techniques via the Internet foster the delivery of highly interactive and individualized interventions to large numbers of people [3].

Computer tailoring — developed even before the Internet period — fosters the development of highly individualized health communication messages that match each person’s unique conditions, characteristics, motivational beliefs (such as knowledge, attitudes, and self-efficacy), and intentions to change and behavior [4]. Internet based, computer tailoring has shown to be effective [5,6] because of working mechanisms such as increasing personal relevance, interest, and frequency of message reading [5,7,8]. Assessment of the reactions of respondents to the CT messages is essential in order to understand their effectiveness.

Rapidly rising skin cancer incidence rates signify the need for effective Internet programs that can contribute to the prevention of skin cancer as well as to education for skin cancer patients [9]. Skin cancer will have a large impact on the demand for health, health care costs and the workload for dermatologists [9,10]. Effective Internet skin cancer programs are available [11,12,13,14]. Assessment of the effects of these programs in subgroups is important given the concerns of potential differential impact of the Internet in groups differing in characteristics such as education and levels of involvement, or relevance of the topic. [1,15,16,17]. For instance, it is conceivable that skin cancer patients are more involved in the issue of skin protection and thus may judge an e-Health program differently than the general population. Given the potential impact of the digital divide, it is important to assess whether Internet approaches are less accepted by groups with a lower education, since this may contribute to increasing health disparities between educational groups [15,16,17,18,19]. Furthermore, it is conceivable that prevention programs may be less appealing to those not at risk. Hence, it is relevant to know whether separate programs for skin cancer patients and non-patients may be needed.

### Objectives

In the present study, we first assessed program evaluations of a new, Dutch, Internet CT program on skin cancer. We next assessed potential differences in program reactions between respondents with a high- and low-educational level, as well as between patients and non-patients, by assessing perceived advantages and disadvantages pertaining to items such as relevance, completeness, and credibility of the CT messages [8].

## Methods

### Design and Procedure

A cross-sectional research design was used, and data collection took place in 2009 from May until August. Skin cancer patients were recruited from a patient database from the Catharina hospital in Eindhoven, the Netherlands. Ethical clearance was provided by the Catharina Hospital in Eindhoven. Patients were sent a letter to their home address with an invitation to participate in this e-study on skin cancer prevention. Additionally, recruitment took place via regional weekly newspapers, social networking sites, and members of an Internet panel. All respondents received a link to complete an online questionnaire about skin protective behavior. Their responses were directly fed into a computer program, which immediately generated personal tailored feedback on-screen.

### Questionnaire

In order to be able to receive CT advice on sunscreen a questionnaire assessed several constructs.

*Personal and predisposing factors *were assessed by questions including gender, year of birth, having children (yes/no), marital status (partner/no partner), education level: low (primary school and vocational education) = 1; high (college and university) = 2, income (above average/below average), and skin cancer history (yes/no). Parental and child skin types were assessed by asking participants to indicate whether they: burn very fast, hardly tan (type 1); burn fast, tan slowly (type 2); do not burn fast, tan easily (type 3); rarely burn, tan easily (type 4); hardly ever burn, tan easily (type 5); or, never burn, easily tan (type 6) [24].

*Suntanning behavior *was measured by three questions. One question measured how often someone goes outside to be in the sun (1 = “never;” 5 = “as often as possible”). The second question measured how long someone was outside on a day off if the sun shone (1 = “never;” 3= “between one and three hours;” 5 = “as long as possible”). The third question measured on what time people are usually exposed to the sun (1 = “never;” 5 = “between 12 P.M. and 3 P.M.”).

*Skin protective behavior *assessed subjective skin protection and objective skin protection. Subjective skin protection was measured by two questions on a 5-point scale asking the participants whether they think they protect themselves properly on the beach or during outdoor activities. (1 = “I always protect myself sufficiently”; 5 = “I never protect myself sufficiently”). Objective skin protection was measured with three questions concerning their suntanning behavior, three questions regarding seeking shadow (“How often are you in the shade between 12 P.M. and 3 P.M.?”), wearing a hat or cap (“How often do you wear a hat or cap on a sunny day?”), and wearing protective clothing (“How often do you wear protective clothing if the sun shines?”). Respondents could answer these questions on a 5-point scale from 1 (“never”) to 5 (“always”).

*Knowledge about skin protection, and skin protection, *was measured with six questions on a three-point scale (1 = “right,” 0 = “wrong” or “don’t know”). Two example questions are: “To protect yourself sufficiently, it is enough to use sunscreen once per day” and “you cannot burn in the shadow?”

*Cues to action *were measured by an index of two questions, assessing: how often someone had seen, heard or read something about skin cancer in the media in the past three months; and how often someone had noticed a change on his or her skin of which he or she thought it might be skin cancer. Both questions used a 5-point answering scale (1 = “never,” 5 = “very often”). Lastly, one question assessed whether the respondent knows people that have or have had skin cancer (1 = “yes,” 2 = “no”). Low reliability on these three questions assessing perceived media messages about skin cancer, perceived skin changes and experience/occurrence of skin cancer in respondents’ social network precluded the formation of one scale.

*Risk perception *was assessed by three questions on a 5-point scale, assessing perceived likelihood, perceived susceptibility, and perceived severity of getting skin cancer. A sum score was made with perceived likelihood and perceived susceptibility (α =.75).

*Attitude *was assessed by 20 items using 5-point scales (1 = “totally disagree”; 5 = “totally agree”); 10 items assessed the perceived pros of sunscreen use (α =.85) and 10 items assessed the perceived disadvantages of sunscreen use (α = .85).

*Social influences *were assessed by nine questions using 6-point scales (α = .78) and assessed the norms about sunscreen use of partner, family and friends, as well as their own sunscreen use and support for sunscreen use.

*Self-efficacy *was assessed by twelve questions using 5-point scales assessing situations in which they may have different levels of confidence regarding the use of sunscreen (α = .75).

*Action plans *were measured with nine statements using a 5-point scale to assess whether respondents made specific preparation plans for sunscreen use (α = .83). Examples are: “I plan to bring a sunscreen with SPF 50+ to places where I plan to stay in the sun for a long time,” “I plan to bring a parasol to, or rent a parasol at, places where I plan to stay in the sun for a long time,” and “I plan to buy sufficient protective clothing and/or a hat or cap.”

*Intention *was measured with three questions using a 5-point scale, with answering categories ranging from 1 (“no, certainly not”) to 5 (“yes, certainly”). The three questions asked whether respondents intended to use sunscreen on a sunny day, use sunscreen every two hours, and use sunscreen 30 minutes before going outside in the sun (α = .86).

### Program evaluation 

Sixteen questions were used to evaluate the tailoring program using strategies from previous studies [8] (also see the results section for a list of all questions in the table). Respondents could indicate their evaluation about the advices on a 5-point scale ranging from 1 (“totally disagree”) to 5 (“totally agree”). Examples of statements are: “the advices were relevant to me,” “the advices stimulated me to improve my behavior,” “the advices were confusing,” and “I missed information in the advices.” Lastly, respondents were asked to give a school grade for all advices ranging from 1 (“very bad”) to 10 (“excellent”). 

### CT program

**Figure 1:**
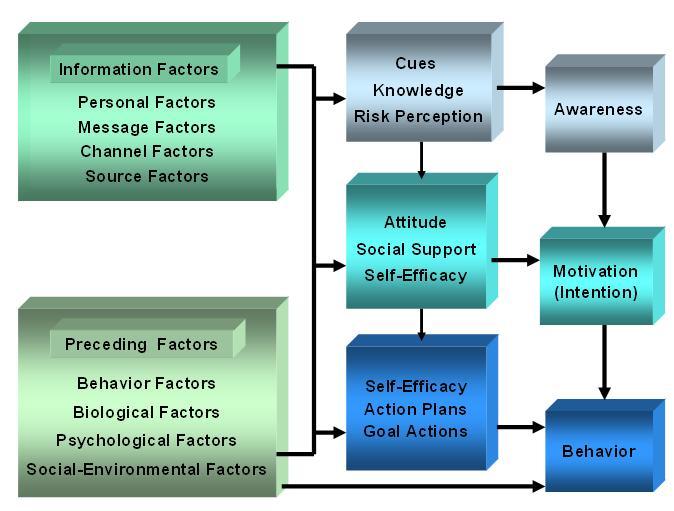
The I-Change Model [8,22].

A first pilot revealed that addressing three behaviors (protective clothing, looking for shade, and sunscreen) would result in too long of a program. This assessment revealed the need to focus on sunscreen use. Next, we assessed the motivational beliefs concerning sunscreen use in the general public and patients and compared the two groups concerning their views regarding sunscreen use. As well, we assessed the factors associated with sunscreen use in both groups using a comprehensive social cognitive model, the I-Change Model [8], postulating that behavior (i.e. sunscreen use) is influenced by action factors (action plans), motivational factors (attitudes, social influence beliefs, self-efficacy), and awareness factors (knowledge, risk perceptions and cues to action) (see Figure 1). The assessment is needed to identify the most important educational needs for program development [20,21].

**Figure 2:**
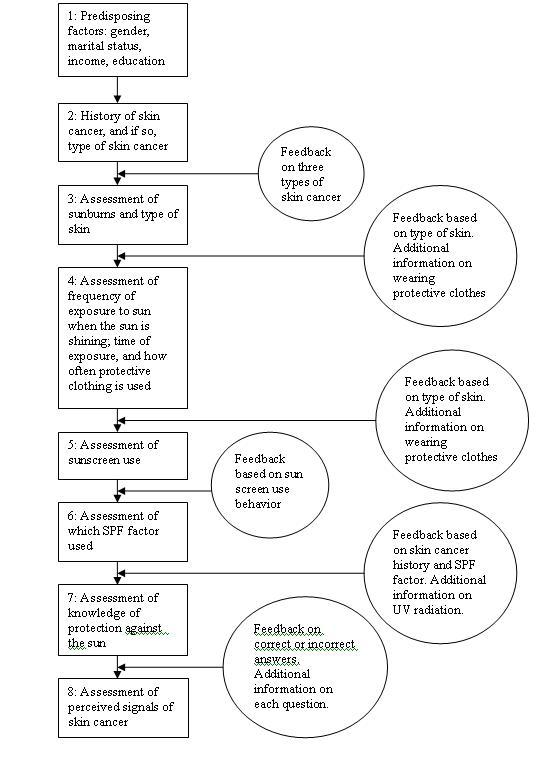
Overview of the CT program.

The new CT program was based on the I-Change model to identify the most important factors to address, and previously conducted strategies and studies on other behaviors that yielded the format that could be used to develop the program [8,25]: such as the utilization of ipsative feedback (Dijkstra & De Vries, 1999), which items to address (van Osch et al., ), and how to use action plans (De Vries et al., 2006;2008; Van Osch et al., 2010). The program provided feedback on the following constructs: sun exposure, sunscreen behavior, type of skin, risk perception, attitudes, social support, self-efficacy, intention and action plans (see Figure 2). A first version was developed and piloted among 11 persons and patients, including one skin cancer expert in order to identify various barriers, such as inconsistencies, unnecessary jargon, and difficult framing. The results were used to improve the final CT program that was used in this study. Results from the pilot as well as from the sample showed that completing the final tailoring program took between 15–25 minutes, including time to read the advices. The time needed did not differ significantly between low- and highly-educated respondents. The process of the CT is summarized in Figure 2, and examples of the CT advices are provided in Table 1.

**Table 1:** Examples of feedback messages

Type of skin feedback	“Your answers show that you are not often in the shade between 12 p.m. and 3p.m., and/or that you do not often wear a cap to protect yourself against the sun. As your doctor may have told you, people with a type 1 skin have the largest risk of getting sunburn. Burning of the skin can lead to getting skin cancer (again). Therefore it is important with your light skin type and skin cancer diagnosis to protect yourself very well against the sun.”
Sunscreen use feedback	“You mentioned that you use sunblock with protection factor 4. This factor (SPF) indicates the protection against UVB-radiation (sunburn). It is also important to know that SPF is universal. Two products with the same SPF (from whatever brand) will offer the same protection. You mentioned before that you have skin type 1, which means that you use a sunblock with an *insufficiently *protection factor**. **Did you know that there are sunblocks that protect you against UVB-radiation as well as UVA-radiation (aging of the skin)? You can see this on the special logo on the label: A circle with the letters UVA in it.”
Attitude feedback	“Your answers show that you see few advantages of protecting your skin. Perhaps you may not know all these advantage. Studies show that if you use proper protection you burn less quickly, and you lower the chance of getting skin cancer. You also lower the chance of getting skin cancer back, in case you have had skin cancer in the past. Furthermore, it slows down the aging of your skin. You will therefore stay young looking for longer, which many people find pleasant.”
Social Support feedback	“Your answers show that you do not receive any social support from your family, because of their opinion and behavior. That is unfortunate, because when people feel supported, it is easier for them to perform a certain behavior. Perhaps you can discuss this topic with your family, so that they can see the benefits of responsible tanning.”
Self-efficacy feedback	“Your answers show that you find it rather difficult to protect yourself when outside. This is understandable, and you are definitely not the only one! It is not always easy to protect yourself as well as possible in various situations. There will always be situations in which it can be difficult to protect yourself. You can prepare yourself for these difficult situations. Some people think about such situations and then come up with a plan to determine how they can protect themselves in these situations. This helps them, and possibly you too? It never hurts to try! Good luck!”
Intention feedback	“You mention that you do not plan on applying sunscreen on a sunny day. Perhaps you find the use of sunblock awkward or uncomfortable? Did you know that there are currently sunscreens on the market that are not sticky? If you really do not want to use sunblock, you can also use different ways of protection, like staying in the shade as much as possible, or wearing protective clothing and a cap. Perhaps you were already planning on this?”

### Statistical analyses

Descriptive statistics were calculated to identify the characteristics of the respondents. T-tests, binomial tests, and chi-squared tests were performed to analyze the demographical differences between the patients and the general population. Analysis of variance (ANOVA) was used to determine the differences between the general population and the skin cancer patients in the program evaluation, and for assessing differences between respondents with a low- and high-level of education. Logistic regression was used to investigate possible confounders for education level and skin cancer history. Linear regression analysis was used to assess the determinants of sunscreen use. All analyses were performed with SPSS 15.0 for Windows. Significant differences are reported when *P *< 0.05.

## Results

### Characteristics of the Sample

After excluding respondents with missing values (>10%), a sample of 387 respondents remained. This sample contained significantly less men (N = 156; 40.3%) than women (N = 231; 59.7%; x^2^= 14.535; df = 1; *P *< 0.001). The characteristics of the overall sample and the subgroups are depicted in Table 2.

**Table 2:** Demographic characteristics of the overall sample, skin cancer patients, general population, and education levels.

		Total group (N=387)	Low education (N=196)	High education (N=191)	Skin cancer history (N=132)	No skin cancer history (N=255)
Gender	Male	156 (40.3)^c^	83 (42.3)^a^	73 (38.2)^c^	40 (30.3)^ c^	116 (45.5)
	Female	231 (59.7)	113 (57.7)	118 (61.8)	92 (69.7)	139 (54.5)
Marital status	No partner	70 (18.1)^c^	29 (14.8)^c^	41 (21.5)^c^	16 (12.1)^c^	54 (21.2)^c^
	Partner	317 (81.9)	167 (85.2)	150 (78.5)	116 (87.9)	201 (78.8)
Children	Yes	256 (68.5)^c^	157 (80.1)^c^	108 (56.5)	105 (79.5)^c^	160 (62.7)^c^
	No	122 (31.5)	39 (19.9)	83 (43.5)	27 (20.5)	95 (37.3)
Skin cancer history	Yes	132 (34.1)^c^	62 (31.6)^c^	70 (36.6)^c^	-	-
	No	255 (65.9)	134 (68.4)	121 (63.4)	-	-
Income	Don’t know	64 (16.5)^c^	36 (18.4)	28 (14.7)^c^	24 (18.2)^c^	40 (15.7)^c^
	< Avg.	86 (22.2)	51 (26.0)	35 (18.3)	21 (15.9)	65 (25.5)
	Avg.	84 (21.7)	47 (24.0)	37 (19.4)	30 (22.7)	54 (21.2)
	> Avg.	153 (39.5)	62 (31.6)	91 (47.6)	57 (43.2)	96 (37.6)
Age		46.2	49.2	43.2	46.3	46.2

^a ^Significant at the .05 level

^b ^Significant at the .01 level;

^c ^Significant at the .001 level

Table 2 also shows that for the group of (former) patients 92 of the respondents were female (69.7%) and 40 were male (30.3%). In the general population 139 (54.5%) respondents were female and 116 (45.5%) respondents were male. Men were on average almost 8 years older than women (50.9 years for men; 43.1 years for women. In the group of (former) patients, most respondents were diagnosed with basal cell carcinoma (N = 65; 49.2%), followed by melanoma (N = 28; 21.2%), and squamous cell carcinoma (N = 10; 7.6%). Twenty-two percent of the respondents (N = 29) did not remember which form of skin cancer they had. Of the total sample, 196 respondents (51%) reported to have a low education.

### Program evaluation

Table 3 shows the overall program evaluation. Concerning the potential advantages of the program, the respondents indicated they find the program informative, complete, personally relevant, that the feedback was well-readable, contributing them to help them to improve their sunscreen behavior, providing nicely arranged information, credible, and that the feedback matched with the answers that they had given. We furthermore assessed potential drawbacks of the program, which were also acknowledged by the respondents, although to a lesser extent than the positive outcomes. Yet, the respondents felt that sometimes the information load was slightly too much and too long, and that information was missing and confusing. Overall, the ratings by all respondents were quite positive, resulting in a 7.78.

**Table 3:** Program evaluation based on education.

		Overall sample	Low education	High education	Sig.^a^
		N=387	N=196	N=191	
**Advantages^b^**					
	Informative	4.19	4.37	4.02	<.001
	Complete	4.12	4.26	3.97	<.001
	Personally relevant	4.11	4.25	3.97	.001
	Well-readable and proper lay-out	4.16	4.25	4.06	.010
	Stimulated to improve sunscreen use behavior	3.81	3.93	3.69	.011
	Nicely arranged	4.30	4.39	4.22	.013
	Helped to improve sunscreen use behavior	3.75	3.86	3.63	.015
	Credible	4.36	4.43	4.29	.026
	I agree with the advices	4.03	4.12	3.95	.051
	Matched my given answers	3.74	3.80	3.69	.245
**Disadvantages ^b ^**					
	Too much information was given	2.22	2.11	2.35	.036
	Too long	2.36	2.27	2.46	.155
	Information was missing	2.36	2.30	2.42	.174
	Confusing	2.01	2.01	2.00	.849
	Grade (1-10)	7.78	7.98	7.58	.001

^a^ Covariate: age

^b^ 1= totally disagree; 5= totally agree

### Differences in Program Evaluation by Low- and High-Educated Respondents

Table 4 depicts the differences between respondents with a low- and high-level of education. Since the two groups differed in their age, we corrected these scores for age. When comparing the two educational groups, Table 2 reveals that respondents with a low education level were significantly more positive about the CT advice than respondents with a high-level of education, resulting in overall scores of 7.98 and 7.58 respectively. Respondents with a low education level were also more positive on most of the advantages of the CT feedback, implying that they found the program more relevant, credible, nicely arranged, informative, well-readable, and complete than those with a higher education. Furthermore, lower educated respondents were more convinced than their higher educated peers that the CT feedback stimulated them to improve their sunscreen behavior. Concerning the disadvantages respondents with a high education level provided, they believed significantly more so, than those with a low education level, that the feedback provided too much information.

### Program Evaluation by the General Public and Skin Cancer Patients

The results of the evaluation of the CT advice by the general public and skin cancer patients are presented in Table 4. Since patients and the general public differed concerning their type of skin and frequency of sun exposure, we corrected the process evaluations for these differences.

**Table 4:** Program evaluation based on skin cancer history.

		Overall sample	Patients	General Population	Sig.^a^
		N=387	N=132	N=255	
**Advantages^b ^**					
	Personally relevant	4.11	4.37	3.98	<.001
	I agree with the advices	4.03	4.23	3.93	.001
	Credible	4.36	4.49	4.29	.004
	Stimulated to improve sunscreen use behavior	3.81	3.99	3.72	.008
	Well-readable and proper lay-out	4.16	4.29	4.09	.016
	Helped to improve sunscreen use behavior	3.75	3.90	3.67	.027
	Nicely arranged	4.30	4.40	4.26	.060
	Matched my given answers	3.74	3.85	3.69	.101
	Complete	4.12	4.18	4.08	.261
	Informative	4.19	4.22	4.18	.676
**Disadvantages^b^**					
	Too long	2.37	2.03	2.53	<.001
	Too much information was given	2.22	1.90	2.39	<.001
	Confusing	2.01	1.79	2.12	.001
	Information was missing	2.36	2.26	2.41	.258
	Grade (1-10)	7.78	8.11	7.61	<.001

^a^ Covariates: skin type and frequency in the sun

^b^ 1= totally disagree; 5= totally agree

Table 4 shows that respondents with a history of skin cancer were significantly more positive about the CT feedback advice than those without a skin cancer history about the advice they received for every single question, with overall scores of respectively 8.11 and 7.61. Patients significantly evaluated the advice more positively than those from the general population for about 9 of the 15 evaluation items. Both groups found the advice equal in terms of being informative, complete, that the advice matched the given answers, and that the advice was nicely arranged.

## Discussion

Our results reveal that the CT feedback was evaluated positively by all respondents, and evaluated the sunscreen CT feedback as relevant, credible, nicely arranged, instructive, complete, felt that the advices matched their answers, helpful, and that the advices stimulated them to improve sunscreen use. The overall score on a scale from 1 (“very bad”) to 10 (“excellent”) was 7.8 indicating a positive evaluation despite these comments made. Suggestions concerning improvements pertained to the fact that sometimes information could be more elaborate; but also that the advices were quite long, and sometimes confusing. In-depth process evaluations are therefore needed to find out which particular program components needed to be improved; and also how to deal with the conflict of adding information on the one hand, and to shorten overall message length on the other [12]. The positive evaluation results are congruent with those reported earlier concerning other health behaviors [8,31,32]. One potential reason for these positive findings may be that program development was based on a combination of approaches implying the utilization of a broad, social cognitive model and principles of communication theories [33,34] and social marketing principles [35], implying that messages were developed and piloted in the target group. Hence, as has been noted before, it is important to acknowledge that besides these positive evaluations, computer technology-based interventions have many advantages when compared to human-delivered interventions. These include lower cost to deliver, greater intervention fidelity, and greater flexibility in dissemination channels, which might include in person (for example, clinic setting), mail, Internet, cell phones, or other delivery channels [36]. CT online interventions utilize a great variety of options for assessing individuals, creating and delivering customized health messages, equipping individuals with the tools necessary to maintain or change their behaviors, and keeping them engaged in their own self-care [5]. Our results support that this strategy is appreciated.

An encouraging finding was that fact that, in contrast to our expectations, respondents of a low level of education evaluated the CT feedback more positively than those with a high level of education, although the latter group was also positive in their evaluation. We would not have been surprised to have seen an opposite result, because the CT feedback implied quite some reading—given the concerns expressed by others concerning Internet use by lower educated groups [15,16,17,35,36]. Yet, the pilots of the program were aimed to identify passages that were difficult and unattractive to read, certainly for respondents with a low level of education. Hence, our results of the program evaluation suggest that this goal has been attained. In our other programs about smoking cessation, physical activity, and nutrition, we also did not find differences in evaluation between the educational groups [8,31]. This suggests that CT feedback can be a very attractive and effective way to reach both low- and high-education level groups.

Patients evaluated the CT feedback slightly, but, significantly, more positively than respondents with no history, who were also very positive concerning the program evaluation. The more positive finding is most likely due to the fact that the topic is more salient to them because of their history of skin cancer. Although they may have received already more information about skin cancer and sunscreen use, they evaluated the feedback very positively. Our findings do not suggest that two separate programs for patients and non-patients are needed.

### Limitations

Our results need to be interpreted with a certain level of caution. First, it is not clear why the low education level group evaluated the program more positively. More qualitative research is needed to asses whether this occurred because the program provided more new information for this group and whether the language used was more adapted to this group. Yet, we did not receive complaints from the high education level group concerning too simply formulated messages. Second, it is difficult to assess how representative the online sample is for the total populations. We also had a relatively large group of highly educated respondents. Yet, our main purpose was not to obtain a representative sample, but to compare the differences in evaluation between high- and low-educated groups. Third, suggestions to shorten the messages were made by all subgroups. These suggestions are relevant to take into account, also within a context that revisits of Internet based programs have found to be quite low, also resulting in high drop-out rates in research studies up to 50% or sometimes higher [37]. A challenge will be to find an optimal balance between length and the provision of essential feedback. Fourth, Internet based methods can use a large variety of behavior change techniques and exposure-promoting elements. In order to enhance exposure, peer and counselor support may result in a longer website visit and that email/phone contact and updates of the website result in more log-ins [3,38]. Recent notions about infodemiology describe important factors related with the distribution and determinants of information in an electronic medium, such as analyzing how people search, navigate, and share information that also yield important insights into health-related behavior [40]. Hence, it is recommended to further extend interventions with these elements. Lastly, an effect evaluation is still needed using a randomized control trial to assess ultimate behavioral effects, as well as analysis may be needed to assess potential differences in program evaluation due to seasonal changes. 

### Conclusions

The most important lesson learned from this study is that CT programs do not necessarily have to be less attractive for lower educated groups. This is encouraging, since this may imply that Internet based interventions can also reach lower educated groups; provided that these interventions are tailored towards the needs and characteristics of lower educated groups. A second lesson learned was that the results of our program evaluation did not suggest a need for the development of separate programs for patients and non-patients.
